# Medical Society of the County of Schuyler

**Published:** 1911-05

**Authors:** John M. Quirk


### SOCIETY PROCEEDINGS

### Medical Society of the County of Schuyler

Reported by JOHN M. QUIRK, M.D., Secretary

On Thursday, March 30, 1911, the Medical Society of the County of Schuyler gave a complimentary dinner at The Glen Springs, Watkins, N. Y., in honor of the fiftieth anniversary of the graduation of Dr. Charles D. Clawson, of Montour Falls, and Dr. Gideon O. Smith, of Odessa. Dinner was served at 2.30, after which Dr. Charles G. Stockton, Professor of the Theory and Practice of Medicine in the University of Buffalo, delivered an address. Dr. Clawson read a paper entitled, Reminiscences of a Student of Fifty Years Ago, and Dr. Smith read a paper entitled, The Recollections of a Young Practitioner Fifty Years Ago. Judge O. P. Hurd, of Watkins, spoke upon, The Experiences of a Patient Fifty Years Ago. Dr. Arthur W. Booth, of Elmira, read a paraphrase on Kipling's Road to Mandalay entitled, The Road to Yesterday. Dr. Sherman Voorhees, Chairman of the Sixth District Branch of the Medical Society of the State of New York, made a few appropriate remarks.

Thirty-four members and guests of the Schuyler County Medical Society sat down to dinner, and Dr. Clawson and Dr. Smith were the recipients of hearty congratulations and expressions of the good will of their friends and neighbors.

Dr. Clawson's paper follows:

### REMINISCENCES OF A STUDENT OF FIFTY YEARS AGO.

In 1860 and 1861 the Medical College of the University of Buffalo was located at the corner of Main and Virginia Streets and just back of the college was the hospital of the Sisters of Charity, at which most of the clinics were held. Occasionally clinics were also held at the General Hospital, where the new Medical College building is now located. The building was a stone structure and at the time was considered as being well adapted to the purpose for which it was used.

The requirements for entering the college, were that "the student must have at least a good, common school education and a certificate of possessing a good moral character."

To enter the graduating class, "he must furnish a certificate showing that he had studied medicine under the instruction of a regularly educated physician at least two years, that he had attended a full course of lectures at an accredited medical college; that he must be at least twenty-one years of age and that the last course of lectures, to complete a full three years, must be at the University of Buffalo. * * * The student must pass a satisfactory final examination in order that he receive a diploma." The method of instruction was by lectures, followed by quizzes by the professors upon the previous lectures.

The class of '61 numbered twenty-four students, and I believe only five are now known to be living. Quite a number entered the Union Army, as in that year the civil war broke out.

The diplomas bear the names of:

Millard Filmore, President of the Board of Curators; Jacobus Platt White, M. D., Professor of Obstetrics and Diseases of Women; George Hadley, M. D., Professor of Chemistry and Toxicology; Thomas F. Rochester, M. D., Professor of the Practice of Medicine and Clinical Medicine; Sandford Eastman, M. D., Professor of Anatomy; Joshua R. Lothrop, M. D., Professor of Materia Medica and Therapeutics; Edward M. Moore, M. D., Professor of Surgery; W. H. Mason, M. D., Professor of Physiology.

As to the individual qualifications of the faculty at the time, I will say that I have been acquainted with the teaching of a number of schools, one before I entered the class of '61 and two others after my graduation and I must acknowledge that, for thoroughness and practical usefulness to the student, the Medical Department of the University of Buffalo has stood among the best.

It must be remembered that great changes have taken place in medical and surgical pathology since 1861, as well as the building up since that time of the science of gynecology and the many improvements in the technic of obstetric practice and surgery; but the students of the school were given the inspiration to do everything in the most improved and approved manner.

As to the individual members of the faculty I believe Professor White was one of the best if not the best teacher in his department at the time in this country. His teaching of the mechanism of labor by the use of a manikin was among the innovations of the time. He had a very large obstetric practice and his practical experience enabled him to demonstrate his subjects so thoroughly that a student attending a case for the first time had confidence to manage every stage of a labor and the subsequent care of the mother. His teaching of the prevention and infectiousness of child-bed fever was not inferior to the teaching of today yet the germ theory of disease was unknown then.

The declaration of Dr. Oliver Wendell Holmes, which was made in 1834, that "child-bed fever is oftener to be attributed to the dirt under the finger nails of the surgeon, than to any other cause," was often referred to by him to indicate to the student that cleanliness was of the utmost importance in the care and management of obstetric cases. His inventive genius gave us an obstetric forceps which has never been improved, but has been imitated without proper credit; and I venture to say that the doctor who owns a White obstetric forceps now would not exchange it for any other on the market. His manner in the lecture room was very pleasant and if a student could not understand his lectures it was a source of much regret to him.

Professor Hadley, as a teacher of chemistry and toxicology, was instructive and entertaining and he made his subject attractive by unvarying success in his experiments before his classes.

Professor Thomas F. Rochester, who was the clinical and didactic lecturer on practice of medicine, was a man of fine ability not only as a teacher but as a diagnostician. His method was rendered impressive by his repetition of important principles and his delivery was distinct and easily comprehended. He had been a resident physician in Bellevue Hospital, New York, for several years and it gave him an experience and practical knowledge which placed him in the front rank as a teacher. While delivering his lectures on malarial fevers, after giving the views of several prominent medical writers, he said: "The theories with reference to the cause of these diseases, and many others, to me is very obscure and unsatisfactory, but I believe the subject will be cleared up by the microscope and physiological research."

This prophesy was fulfilled about the year 1880.

Professor Sandford Eastman, lecturer on anatomy, was a native of my own county (Seneca). Although his subject was generally considered dry he succeeded in interesting his classes so that the students passed good examinations.

Professor Joshua R. Lothrop, lecturer on materia medica and therapeutics, was ahead of the times in teaching his subject. He gave particular attention to the indications of remedies and the physiological action of drugs, thus laying the foundation for future study by the student.

Professor Edward M. Moore, the lecturer on surgery, had a national reputation as a skilled operator. Among the important subjects which he taught was the manipulative method of reducing dislocations. At that time an efficient hemostatic forceps was scarcely known, and an operator's skill was measured by his intimate knowledge of anatomy and the avoidance of arteries to prevent loss of blood. I saw him make several operations for stone in the bladder without the loss of an ounce of blood. He was the inventor of a lithotrite which has been the model for nearly every instrument used to crush urinary calculi. He was very successful in his operations notwithstanding the fact that aseptic and antiseptic surgery was unknown. In his teaching he distinctly warned us that in the majority of cases succeeding an operation, if extensive, we might expect surgical fever. He always, however, insisted upon surgical cleanliness and many of his operations were not followed by surgical fever. In such cases his common expression was "we are getting union by first intention."

About sixteen years later I took a case to him for the removal of a bullet from the hand of a young man who was accidentally shot. After quite a severe operation under chloroform he said: "I will dress this antiseptically;" which consisted in the application of a 3 per cent. solution of carbolic acid applied upon lint and covered with a bandage.

Professor W. H. Mason, then a young man, was the teacher of physiology and had entered upon the duties of his department that same year, 1861. He had spent three years in Europe and his teaching was up-to-date. He was very enthusiastic in his studies and experimental researches and he became very popular as a teacher and lecturer.

Today the Buffalo Medical College is a great institution and has a brilliant record. The new buildings and equipment are well adapted to the needs of its advanced teaching. Its high standards place it among the best schools of this country. The clinical advantages of the school are all that could be desired and its location in the city of Buffalo is fortunate as to geographical position. It numbers among its faculty some of the most progressive men of the age. The changes which have occurred and the new sciences, we might call them, which have been developed and have contributed to the building up of the practice of medicine and surgery since the 60's are truly marvellous.

Then there were only two books published in the English language on diseases of women: one by Megs of Philadelphia, and another by Bedford of England. It is needless to say that these books are now kept with our curios and our ancient literature.

In those days the peritoneal cavity was considered surgically as a domain, whose borders could not be invaded without inviting death. Peritonitis which we now know, in a large proportion of cases to originate in the appendix, was almost invariably a fatal disease and not more than 10 per cent. of the cases recovered.

The whole subject of bacteriology as a collateral science to the practice of medicine has been developed since 1875.

If medical science should advance in the next fifty years as rapidly as it has in the past half century, the golden period of its becoming an exact science would indeed be dawning.

Dr. Smith's paper follows:

I will begin with a few brief cases of my third year of medical student life. I had previously taken a course of lectures at the Buffalo Medical School under the instructions of Drs. Sanford Eastman, Professor of Anatomy, Thomas F. Rochester, Professor of the Principles and Practice of Medicine; Frank H. Hamilton, Professor of Surgery; James P. White, Professor of Obstetrics; Austin Flint, Jr., Professor of Physiology and George Hadley, Professor of Chemistry. It is superfluous to say these were brilliant teachers. Peace be to their ashes. I studied medicine with Dr. Lyman Congdon, a graduate of Buffalo University. After my first course of lectures I rode with him occasionally visiting the sick. He took me to see a young man who while picking apples had fallen about twelve feet from a tree and struck square upon his feet. Soon he became faint and had to lie down and the doctor was sent for; found patient with a weak pulse and some pain in the right side; gave a mild anodyne. That night our patient died. Next day a postmortem was held and the abdomen was found distended with blood; on removal of which we discovered the right lobe of the liver cracked open three inches in length caused by the concussion of the man striking on his feet.

While attending lectures the next winter at Buffalo, I described the case to Professor Rochester without giving him the findings of the postmortem and he readily stated what we found as he had had a similar case. One evening my preceptor drove up to the office with something on his mind that worried him. I asked him what was the matter. He said he had just left a big burly Irishman with a large hernia out, with a large dose of opium in him and hips elevated in hopes the hernia would go back during the night. As I had seen something of the kind in the hospital I said I guessed not; and if he would take me in the morning to see the case with him and take along a good bottle of chloroform, I guessed we would get it back. Bright and early the doctor came to my boarding place with his rig and old balky mare and armed with a bottle of chloroform we were soon at the bedside of the Irishman. We found a man with a large nasal proboscis and the last half inch turned up. I readily perceived that nose meant business when we come to give the chloroform. The hernia by this time had assumed quite large proportions. The doctor began giving the chloroform and I was to reduce the hernia by taxis. Pretty quick, as I had expected, the fun was on. The chloroform began to work and so did that nose, and our Irishman declared he was not going to snuff any more of that "damned stuff." Fortunately my perceptor was a power and weighed two hundred pounds. I motioned to him to reach over the low head board and grab him and hold him down to the bed while I gave him the chloroform. He did so and as our patient consigned us to the lower regions, he inhaled the chloroform finely and we soon had him snoring lustily while I reduced the hernia.

My next case of student life was one day with my preceptor to see a case, where a man about 60 had had a shock and was fast lapsing into ramollissement. The doctor asked me to make the prescription. I suggested tonic doses of quinine and nux vomica given after meals. One of the family came hurriedly to the office in a few days and wanted some more of the medicine just like we left when there and said it was curing him. The prescription was continued. The man began to pick up, got up from a bed he had occupied several weeks and went off to Pennsylvania farming.

I returned that fall and continued my studies and lectures at Buffalo, received my diploma February 21, 1860, and commenced the practice of medicine April 1, 1860. I encountered a terrible scourge of diphtheria in 1862-3 and treated nearly a hundred cases in those two years and I followed at that time Austin Flint who was considered the best authority on the practice of medicine at that date. I think it was the consensus of medical opinion that we had no better practitioner of medicine in the world at that time. His treatment was sulphate of quinine, tr. of the chloride of iron, and the chlorate of potassa. My sheet anchor where children were old enough was twenty grains a day of the sulphate of quinine. I lost but few cases where twenty grains a day gargled and swallowed were used. Where the disease involved the larynx, the trachea, the bronchi and their bifurcations, the patient was as good as dead. I fought such a case desperately and thought if I could only throw the membrane up with an emetic, I might save my patient. I tried it once to see what I could do and in the act of vomiting, I obtained a membrane a perfect cast of the trachea and the right bronchia. The cast of the right bronchia had attached the casts of thirteen of its bifurcations over an inch long. I exhibited it to this County

Medical Society at that time. and gave it to Professor Nelson Nivisin, of Burdett, who lectured on pathology in the Syracuse Medical College. I have treated many cases of appendicitis of the present day as cases of peritonitis and cured a good percentage of them with opium sufficient to subdue all pain and if necessary given within an inch of the patient's life. One patient had what we would now call suppurative appendicitis. This patient was the father of Professor L. H. Bradley the optician of Watkins. Edwin Bradley was taken with pain and tenderness thirty-five years ago at what we now call McBurney's point. There was high fever. Soon the abdomen became red, shiny and springy on palpation. There was pus there. I asked Dr. E. B. Wager, of Montour Falls, to see the case with me. We both decided pus was present. We introduced a grooved exploring needle and withdrew it with the groove filled with pus. I again introduced the needle and cut down on the groove and discharged quite a quantity of pus. This patient recovered. I can now see we built better than we knew and opened an appendix abscess. These cases of peritonitis I have held solid under opium for two weeks or more, without moving the bowels all that time until all inflammation had subsided when without physic or enemas the bowels would move of their own accord regularly and naturally.

The friends in one case that appeared to be doing well under my care on good size doses of opium took it into their heads that a consultation must be held and Dr. J. B. Ames was called. He said to the friends that the bowels should be moved with physic at once and should have been moved every day. He prepared a good big dose of calomel and gave the patient who in twenty-four hours was a corpse.

Over thirty years ago I had another case of suppurative appendicitis. This was a young lady aged twenty-four, with fever, pain and tenderness over McBurney's point. I kept the pain down with a plenty of opium, fed her meat extracts and broths. One day all of a sudden she had to get on the vessel and nearly a pint of pus was delivered per rectum into the vessel. Nature here operated safely for appendicitis.

In 1863 on the 14th day of January, I was called four miles in deep snow to see a case of pneumonia. I found a young man twenty-one years old desperately sick with the right lung completely filled and a part of one lobe of the left lung also. He was cyanosed and it did not seem that he would live twenty-four hours. His lips and nails were purple. He was in a small seven by nine bedroom joining a large sitting room filled with friends, robbing him of the air he was desperately struggling to get. The first thing for me to do was to give this young man more oxygen. We could not buy it those days and we had to have it. Where could we get it? We could at least give him plenty of God's fresh air. We soon emptied that sitting room of the friends; had a bed put up in the middle of the room and the patient in it. He still kept cyanosed and I began to think that the undertaker would soon be around. Our patient had to have more and better air if he lived. The fire was put out; my patient did not need any fire as it was robbing him of what little oxygen the room contained, and his temperature was 104° or 105°. When the fire was put out, he grew less cyanosed. Everybody was ordered into the kitchen, and they were willing and glad to go out of the cold sitting room where the patient was. A relay of nurses every hour with overcoats or shawls on was ordered from the kitchen only one at a time, to take care of him. If more than one person was in the room at a time with him, he would become cyanosed. When I made my visits the nurse retired to the kitchen. Small doses of aconite and digitalis and when bloody expectoration was profuse a little ergot constituted the principal medical treatment. My patient recovered and now resides in Michigan, a prosperous farmer.

A Mrs. Johnson, age thirty-two years, mother of four children and pregnant four months had been in good health previous to this accident and had been about the house the day before. She awoke about one o'clock A. M., August 4, 1871, with severe pain in the pit of the stomach. She called up her husband who slept below with some of the children and asked him to get her some peppermint which he did. At 7 A. M. I was called; found general tenderness over the abdomen and supposed I had a case of peritonitis to treat. Her pulse was 90, rather weak. Prescribed anodynes and a fomentation and left intending to call again during the day. I was recalled at 1 P. M. in haste and found my patient pulseless at the wrist and about dying. There was no external flowing. Patient said she had not had anything done to produce abortion and had not been hurt. This answer was given after I had informed her she was dying. The patient expired at 1.30 P. M. before her near friends arrived. Soon the friends came in and I was closely questioned by her brother and others in regard to the cause of death. I unhesitatingly informed them I did not know. I reported the case to Professor James P. White, Professor of Obstetrics in Buffalo Medical College, with other information in regard to the case. He said: "I congratulate you on possessing the courage to say you did not know the cause of death; and further, that we should make far less blunders if we would resolve not to profess to a degree of knowledge which we do not possess." I asked for a postmortem which was readily granted by the friends. I immediately invited Drs. Bailey, Wager, Ames and Beach to assist me and in the presence of many friends and relatives, the abdomen was opened at 5 P. M. of that day. We found the abdomen filled with blood; on removing which, and examining for the cause, the uterus was found ruptured on the left side and the head of a fetus protruding into the abdomen. The mystery was unveiled and public opinion changed from the verdict that Johnson had poisoned his wife, to one founded on facts derived from a very satisfactory postmortem.

Dr. Booth's poem was read as follows:

By the dear old lake of Erie there I now
would love to be,
Where my Alma Mater's sittin' an'
I know she thinks of me.
For the wind is still a blowin' and the
college bells they say,
Come you back ye ancient scholars
To your University.
On the road to yesterday
Where we used to work and play,
Can't you 'ear the students yelling
Buffalo is great today.
On the road to yesterday
At the University.
And the past comes up like thunder
outer
Memories dear today.

'Er Faculty was noted for its knowledge
vim and go,
An' 'er name was ever famous just the
same as Buffalo,
An' 'twas there I fust was smokin'
of a whacking big cheroot
And wondering if I ever would get
over last night's toot.
Bloomin' students dull as lead,
Never worked but loafed instead,
Plucky lot we knew of medicine but I hoped
we'd get ahead.
Oh; those days of yesterday
At the University,
'Ow we had to work like thunder
For the graduation day.

But that's all shoved behind me—
Long ago and far away,
An' there ain't no Profs a tellin'
Aconite will save the day.
An' I'm learnin' 'ear in Watkins
What the ten year doctor tells,
That a mustard foot bath's better for
Pneumonia and all ills.
No you won't need nothin' else,
But them spicy mustard smells,
And the fresh air and the diet
And the nussing by the gels
Off the road to yesterday,

Leucocytes have come to stay,
An' there ain't no use in talkin'
I'm clean lost upon the way.

Ship me somewhere back of sixty
Where the best was like the worst,
An' the man who drove the hardest
got to treat the case at first—
For the dear old code of ethics
with its fine distinctions clear,
Was simply used to guide the few
Who held traditions dear.
On the road to yesttrday,
Where our Masters led the way.
With their purging blue mass doses
And their lancet to the fray.
On the road to yesterday,
Where we fellows fain would stay
For our minds are stunned
by Thunder!
At the change there is today.

